# Anti-HLA and Anti-MICA Antibodies in Liver Transplant Recipients: Effect on Long-Term Graft Survival

**DOI:** 10.1155/2013/828201

**Published:** 2013-11-24

**Authors:** Michał Ciszek, Bartosz Foroncewicz, Krzysztof Mucha, Dorota Żochowska, Bogna Ziarkiewicz-Wróblewska, Marek Krawczyk, Leszek Pączek

**Affiliations:** ^1^Department of Immunology, Transplantology and Internal Diseases, Medical University of Warsaw, Nowogrodzka Street 59, 02-006 Warsaw, Poland; ^2^Department of Anatomic Pathology, Medical University of Warsaw, Chałubińskiego 5, 02-004 Warsaw, Poland; ^3^Department of General, Transplant and Liver Surgery, Medical University of Warsaw, Banacha 1A, 02-097 Warsaw, Poland

## Abstract

*Objective*. Presence of anti-HLA antibodies has a well-known impact on kidney grafts survival; however their role in liver transplantation has not been fully elucidated. We conducted a 7-year prospective study to show correlation between presence of anti-HLA and anti-MICA antibodies and liver graft survival. *Methods*. Blood samples from 123 liver transplant recipients were collected during patients routine visits. Time from transplantation to blood sample collection was different for each patient. Blood samples were tested for anti-HLA (separately class I and II) and MICA antibodies using Luminex assays. *Results*. There were 32 (26%) patients with positive anti-HLA and 37 (30%) with positive anti-MICA antibodies. Graft loss occurred in 7 cases (23%) in anti-HLA positive group compared to 20 (22%) in anti-HLA negative group (*P* = ns) and in 8 cases (22%) in anti-MICA positive group but 19 (23%) in anti-MICA negative group (*P* = ns). No correlations were detected between presence of antibodies and acute graft rejection (AGR). Presence of any antibodies (anti-HLA or anti-MICA antibodies) correlated with late graft rejection (*P* = 0.04). *Conclusion*. Presence of anti-HLA or anti-MICA had no impact on long-term liver graft survival; however, detection of any antibodies was correlated with episodes of late graft rejection.

## 1. Introduction

Presence of donor specific anti-HLA antibodies (DSA) is a negative predictor of graft survival in kidney transplantation. Moreover, the presence of any anti-HLA antibodies without accessing their specificity increases the risk of kidney graft failure [1]. Transplanted liver is considered a less “immunogenic” organ than kidney. Antibody mediated rejection (AMR) of liver graft has not been considered an important pathology in ABO compatible, cross-match negative liver transplantation for many years. Moreover, there are no clear histopathological criteria of AMR diagnosis in liver graft and no consensus about the value of vascular C4d deposits [2]. However, evidence for pathological role of high-titre antibodies to class I antigen in the vanishing bile duct syndrome after liver transplantation was described by Donaldson et al. 25 years ago [3]. Moreover, presence of preformed antibodies against donor HLA antigens detected by cytotoxic assay or multibead array was associated with decreased 1- and 5-year liver graft survival [4]. AMR cases in AB0-compatible, cross-match negative liver transplants with presence of anti-HLA antibodies have been described with graft function improvement after therapeutic depletion of anti-HLA antibodies titer [5, 6].

There are conflicting data regarding impact of anti-MICA antibodies on acute rejection episodes and kidney graft survival [7, 8]. The role of anti-MICA antibodies in liver transplant was investigated in only few studies. Biliary cast syndrome was diagnosed in 34.4% liver transplant recipients who had posttransplant high serum level of soluble form of MICA (sMICA) compared to 17.3% in recipients with normal sMICA level [9]. However, no association between MICA cell surface expression in liver biopsy sections and presence of serum anti-MICA antibodies and episodes of acute rejection was observed in a study of 84 liver transplant patients [10].

The 14th International HLA and Immunogenetics Workshop Prospective Chronic Rejection Project was an international collaborative study of 45 transplant centers to assess impact of anti-HLA and anti-MICA antibodies detected after transplant on chronic graft failure. The 1-year follow-up data published in 2007 showed significant impact of anti-HLA antibodies on kidney graft survival [1]. Data concerning long-term liver graft survival has never been published.

We present 7-year follow-up data and in-depth clinical and pathological analysis of 123 liver transplant recipients from our center participating in this project.

## 2. Material and Methods

### 2.1. Patients

Blood samples were collected from 123 liver transplant recipients who were at least 6 months posttransplant between September and November 2005 during the patients' routine visits in the Outpatient Department of Transplantation Institute Medical University of Warsaw (MUW). Time from transplantation to blood sample collection was different for each patient. Liver transplantations were performed in the Department of General, Transplant and Liver Surgery MUW, between June 1999 and January 2005 in most cases. Only 4 transplantation cases were performed earlier: 1 in 1995, 1 in 1996, and 2 cases in 1997. All liver transplants were blood compatible but were performed without preoperative cross-match test. Patient death, graft failure, clinical and biopsy-proven rejection episodes, and liver function tests were recorded prospectively during the 7-year follow-up period. Causes of patients' deaths were arbitrary divided as “nonimmunological” (cancer, cardiovascular diseases, and HCV related cirrhosis) and “immunological.” Retrospective data concerning liver disease before transplantation, clinical and biopsy proven rejection episodes before entrance to the study, type of immunosuppression (basiliximab induction, type, and number of immunosuppressive drugs), and HBV and HCV infection status were taken from patients' medical records. Liver diseases before transplantation were arbitrary divided as “immunological” (autoimmune hepatitis, primary sclerosing cholangitis, and primary biliary cirrhosis) and “nonimmunological.” Acute graft rejection episode during first 6 months after transplantation was categorized as early rejection. Clinically suspected acute graft rejection episodes were diagnosed based on sharp elevation of liver enzymes which normalized after treatment with methylprednisolone pulses and/or with an increase in tacrolimus dose. HBV infection was diagnosed on the basis of repeated presence of anti-HBc antibodies and HCV infection diagnosis was based on positive anti-HCV antibodies immunoenzymatic assay.

### 2.2. Laboratory Analysis

Blood samples were tested for the presence of anti-HLA class I and II antibodies using Luminex kits (One Lambda, Inc., Canoga Park) and anti-MICA antibodies were tested using Luminex assays with MICA *001, *002, *004, *007, *012, *018, *019, and *027 antigens purified from recombinant cell line coated onto Luminex beads in Terasaki Foundation Laboratory (Los Angeles, CA). Mean fluorescence intensity (MFI) 1500–2999 was considered weak positive and 3000 or greater strong positive for MICA 001–027 antigens, except for MICA 019 antigen (weak positive 2500–3999 and strong positive 4000 or greater).

### 2.3. Statistical Analysis

Statistical significance of differences among groups was assessed by chi-square or Fisher's exact test. Additional analysis of the odds ratios was done by logistic model. The Wilcoxon rank-sum test was used for analysis of nonnormal data. *P* values of less than 0.05 were taken as significant.

## 3. Results

Anti-HLA and anti-MICA antibody results and full medical reports were available for 123 liver transplant recipients (61 women, 62 men). Three patients were lost to followup and were not included in 7-year survival analysis. Mean age at entry to the study (day of blood sample collection) was 44 (19–68) years and mean time between liver transplantation and blood collection for anti-HLA and anti-MICA antibodies was 34 (7–174) months (1 patient 125 and 1 patient 174 months). Primary liver diseases were hepatitis C (HCV) in 29 cases, toxic liver disease in 19, autoimmune hepatitis (AIH) in 12, primary biliary cirrhosis (PBC) in 12, primary sclerosing cholangitis (PSC) in 10, hepatitis B (HBV) in 9, and others in 32 cases (Wilson disease, Budd-Chiari syndrome, hemochromatosis, and cholangial malformations). Patient characteristics are listed in Table 1.

### 3.1. Anti-HLA Antibodies

**Figure 1:**
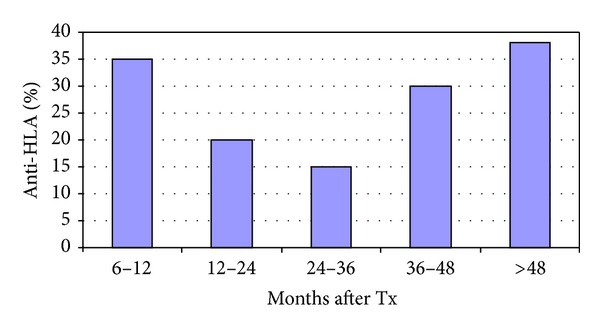
Correlation between presence of anti-HLA antibodies and time after liver transplantation.

Thirty-two patients had titers of anti-HLA antibodies (8 with both classes I and II, 16 with only class I, and 8 with only class II). Mean age of patients at the time of blood collection in anti-HLA positive group was 43 (21–60) years versus 44 (19–68) years in anti-HLA negative group and time after transplantation was 32 (7–101) months and 35 (8–174) months, respectively (*P* = ns). Positive correlation between percentage of patients with positive anti-HLA, but not anti-MICA antibodies, and time after transplantation was observed in groups after 2nd posttransplant year. Anti-HLA antibodies were present in 7 from a group of 20 patients (35%) at 6–12 months after transplantation, 7 from 35 patients (20%) at 12–24 months, 4 from 27 patients (15%) at 24–36 months, 6 from 20 patients (30%) at 36–48 months, and 8 from 21 patients (38%) >4 years after transplantation (Figure 1). No significant correlations were observed between presence of anti-HLA antibodies (anti-HLA I and anti-HLA II nor separately) and the following variables: patients' age and sex, time since liver transplantation to blood collection, primary liver disease (both “immunological” and “nonimmunological”), HBV and HCV infection, basiliximab induction, or immunosuppressive drugs (both type and number). 

### 3.2. Anti-MICA Antibodies

Thirty-seven patients with anti-MICA positive antibodies included 10 patients with weak positive and 27 patients with strong positive antibodies. Mean age of patients at the time of blood collection was 44 (20–68) years in anti-MICA positive group versus 42 (19–68) years in the anti-MICA negative group (*P* = ns) and time after transplantation was 43 (11–174) months and 30 (7–125) months, respectively (*P* = 0.02). Presence of anti-MICA antibodies (both all positive and only strong positive) did not significantly correlate with the following variables: patients' age and sex, time since liver transplantation to blood collection, primary liver disease (both “immunological” and “nonimmunological”), HBV and HCV infection, basiliximab induction, or immunosuppressive drugs (both type and number).

### 3.3. Patient and Graft Survival

**Figure 2:**
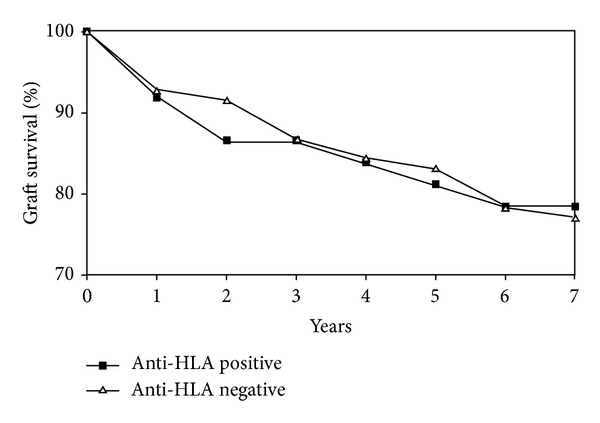
Survival of patients in anti-HLA positive and anti-HLA negative groups. Graft loss occurred in 7 (23%) patients in the anti-HLA positive group and 20 (22%) in the anti-HLA negative group (*P* = 0.79, OR = 0.76 [0.26–2.25]).

**Figure 3:**
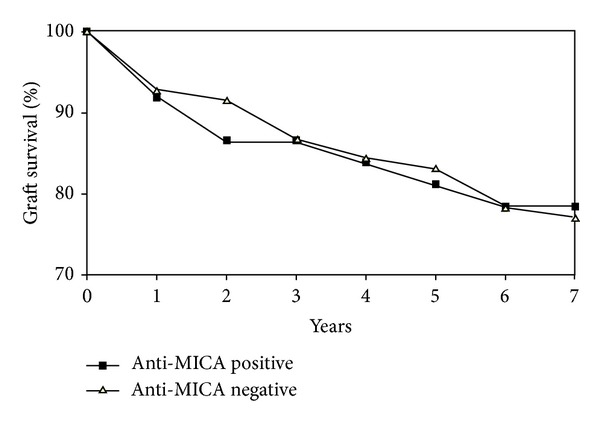
Survival of patients in anti-MICA positive and anti-MICA negative groups. Graft loss occurred in 8 patients in anti-MICA positive group (22%) and 19 (23%) in anti-MICA negative group (*P* = 0.86, OR = 1.03 [0.38–2.76]).

Twenty-seven patients died during the 7-year study period. Progressive graft failure was the main cause in 16 cases whereas other medical conditions like malignancies, neuroinfection or cardiovascular disorders were the main cause of mortality in 11 patients. No retransplantations were performed in this group during the study period. The only predictors of longer patients survival in the whole group were younger age at transplantation (*P* = 0.008) and immunosuppression with tacrolimus (*P* = 0.049, OR = 2.86 [1.07–7.62]) and 15 of 93 (16%) patients died in tacrolimus group in comparison to 12 of 27 patients (44%) in nontacrolimus group. Graft loss occurred in 7 (23%) patients in the anti-HLA positive group and 20 (22%) in the anti-HLA negative group (*P* = 0.79, OR = 0.76 [0.26–2.25]). Presence of anti-HLA antibodies was not a significant predictor of patients and grafts survival in analyses of the whole group or separately in anti-HLA I and anti-HLA II positive groups (Figure 2). Graft loss occurred in 8 patients in anti-MICA positive group (22%) and 19 (23%) in anti-MICA negative group (*P* = 0.86, OR = 1.03 [0.38–2.76]) (Figure 3). Presence of anti-HLA or anti-MICA antibodies was also not a predictive factor of graft failure in an analysis which excluded the 11 patients with known “nonimmunological” cases of death. No significant correlation was detected between patients' survival and the following variables: time since liver transplantation to blood collection, primary liver disease (both “immunological” and “nonimmunological”), HBV and HCV infection, or basiliximab induction.

### 3.4. Acute Graft Rejection

Biopsy-proven early AGR were diagnosed in 23 cases and early AGR was clinically suspected in 24 cases based on retrospective data. Basiliximab induction was a negative predictor of both all and biopsy-proven cases of early AGR (*P* = 0.03, OR = 2.56 [1.14–5.74] and *P* = 0.005, OR = 5.26 [1.63–17.03], resp.). HCV infection was a significant negative predictor of all AGR (*P* = 0.04, OR = 2.74 [1.11–6.77]) but not biopsy proven early AGR cases. Early AGR was not significantly correlated with patients age and sex, primary liver disease (both “immunological” and “nonimmunological”), HBV infection, nor number or type of immunosuppressive drugs. Late biopsy proven AGR was diagnosed in 13 cases and histopathological findings of concomitant chronic rejection were found in 4 (31%) cases. Late AGR did not significantly correlate with patients age and sex, time since liver transplantation to blood collection, primary liver disease (both “immunological” and “nonimmunological”), HBV and HCV infection, induction with basiliximab, nor number or type of immunosuppressive drugs.

### 3.5. Acute Graft Rejection and Anti-HLA/MICA Antibodies

No correlations were detected between presence of anti-HLA antibodies (neither all cases nor HLA I and HLA II separately) or anti-MICA antibodies (neither positive nor only strong positive cases) with early or late AGR. In contrast, presence of any antibodies (anti-HLA or anti-MICA antibodies) correlated with late AGR (*P* = 0.04, OR = 4.03 [1.05–15.65]).

### 3.6. Liver Function

Liver function test was performed every 3 months during patients' routine visits in Outpatients Department. The anti-HLA positive versus anti-HLA negative groups did not differ in serum bilirubin concentration, alanine aminotransferase (ALAT), or gamma-glutamyl transpeptidase (GGTP) activity at the end of the 7-year study. Similarly, these variables did not differ between the anti-MICA positive and anti-MICA negative groups. However, mean bilirubin serum concentration was significantly higher in a group of patients with late acute graft rejection (*P* = 0.02) than that in patients with no AGR.

## 4. Discussion

We analyzed the impact of anti-HLA and anti-MICA antibodies on liver graft failure and function at the 7-year followup and also the correlation between presence of these antibodies and several clinical and pathological data in 123 AB0 compatible liver transplant recipients. 

Anti-HLA antibodies may be detected in early period after liver transplantation due to massive blood transfusion usually required during surgical procedures, so patients at least 6 months after operation were included in this study. Moreover, the percentage of patients with positive anti-HLA antibodies changed in a time-dependent manner in study group, suggesting that we observed true recipients' humoral reactions to donor antigens rather than presence of preformed antibodies. Taner et al. showed that preformed donor specific antibodies (DSA) tend to disappear after liver transplantation in most cases but if persist complement activation in the liver allograft could be found in biopsy but it did not impact graft function at 1-year after transplantation [11]. Our data confirmed the following previously reported association: lower incidence of early acute graft rejection with basiliximab induction [12] and longer survival in patients receiving tacrolimus-based therapy [13], suggesting that data from our study could be representative for most liver transplant recipients. 

In our analyses, no significant correlations were detected between presence of anti-HLA/anti-MICA antibodies and factors which could potentially activate humoral immune system response, like primary autoimmune liver diseases, minimalization of immunosuppression (HCV and HBV infection or HCC in explanted liver), or type and number of immunosuppressive drugs. The presence of anti-HLA (generally and separately in HLA I and II classes) and anti-MICA antibodies did not significantly correlate with 7-year graft survival or acute graft rejection in either early or late posttransplant period. In contrast, the presence of any antibodies (anti-HLA or anti-MICA antibodies) significantly correlated with late graft rejection. This observation suggests that increased activation of humoral immune system could be involved in late rejection episodes and agrees with results of a prospective study showing that DSA were found significantly more in liver transplant recipients with biopsy proven chronic rejection (36 of 39 (96%)) than in patients without rejection (24 of 39 (61%); *P* = 0.003) and that patients with chronic rejection had a higher mean fluorescence intensity (MFI) of DSA than comparator patients [14]. In another study, circulating DSA and diffuse portal complement C4d deposits were associated with steroid resistant liver graft rejection and were found in 60% (6 of 10) of patients with ductopenic rejection. Humoral alloreactivity frequently appears to co-occur with cellular mechanisms of rejection in AB0-compatible liver transplantation and plays a role in ductopenia development [15]. Monitoring of liver graft function is difficult because no single test (like creatinine clearance in kidney transplantation) correlates with liver graft function. Liver enzymes activity in serum changes over time and usually declines in advanced liver failure. Typical indicators of hepatocytes failure, like hypoalbuminemia or coagulopathy, are helpful only in diagnosis of the late phase of liver insufficiency. High serum bilirubin concentrations can be a good indicator of liver graft function if significant extrahepatic bile duct stricture is excluded. We found that serum bilirubin concentrations were significantly higher in patients with late graft rejection. Intrahepatic cholestasis is a typical finding in chronic liver graft rejection leading to graft failure and histological findings of chronic rejection were present in 4 of 13 (31%) biopsy proven cases of late acute rejection in the study group. 

Limitations of this study included that antibodies were screened only once and at different time points after-transplantation for each patient in the study group; however it was caused by the methodology of the Workshop. Secondly, the presence of anti-HLA antibodies generally, but not DSA, was tested; however, availability of single-antigen Luminex assays was limited when the study was started. We are planning to reevaluate remaining patients sera for the presence of DSA.

## 5. Conclusions

Presence of anti-HLA or anti-MICA antibodies did not significantly correlate with inferior graft survival nor with increased incidence of acute graft rejection. In contrast, higher humoral immune system activity defined as presence of any antibodies (anti-HLA or anti-MICA antibodies) was a significant predictor of late graft rejection. 

## Figures and Tables

**Table 1:** Patients characteristics.

	Anti-HLA positive	Anti-HLA negative	Anti-MICA positive	Anti-MICA negative
Number	33	90	37	86
Mean age (years)	47	47	44	47
Sex F/M	16/17	42/48	18/18	41/46
Primary liver disease*				
(i) AIH	5	7	5	7
(ii) PBC	4	8	3	9
(iii) PSC	2	8	2	8
(iv) HCV	7	22	8	21
(v) HBV	2	7	2	7
(vi) Toxic	6	13	4	15
(vii) Other	7	25	12	20
Mean time since LT to blood collection (months)	24	26	34	25

*AIH: autoimmune hepatitis; PBC: primary biliary cirrhosis; PSC: primary sclerosing cholangitis; HCV: hepatitis C virus; HBV: hepatitis B virus, LT: liver transplant.
